# A Parallel Reconstruction Scheme in Fluorescence Tomography Based on Contrast of Independent Inversed Absorption Properties

**DOI:** 10.1155/IJBI/2006/70839

**Published:** 2006-12-14

**Authors:** Xiaolei Song, Ji Yi, Jing Bai

**Affiliations:** Department of Biomedical Engineering, Tsinghua University, Beijing 100084, China

## Abstract

Based on an independent forward model in fluorescent tomography, a parallel reconstructed scheme for inhomogeneous mediums with unknown absorption property is proposed in this paper. The method considers the two diffusion equations as separately describing the propagation of excited light in tissues with and without fluorescent probes inside. Then the concentration of fluorophores is obtained directly through the difference between two estimations of absorption coefficient which can be parallel inversed. In this way, the multiparameter estimation problem in fluorescent tomography is transformed into two independent single-coefficient determined schemes of diffusion optical tomography (DOT). Any algorithms proved to be efficient and effective in DOT can be directly applied here. In this study the absorption property is estimated from the independent diffusion equations by a gradient-based optimization method with finite element method (FEM) solving the forward model. Simulation results of three representative occasions show that the reconstructed method can well estimate fluorescent property and tissue absorption distribution.


Hindawi Publishing Corporation
International Journal of Biomedical Imaging
Volume 2006, Article ID 70839, Pages 1–7
DOI 10.1155/IJBI/2006/70839

### A Parallel Reconstruction Scheme in Fluorescence Tomography Based on Contrast of Independent Inversed Absorption Properties

**Xiaolei Song, Ji Yi, and Jing Bai**

*Department of Biomedical Engineering, Tsinghua University, Beijing 100084, China*

Received 21 February 2006; Revised 5 June 2006; Accepted 13 August 2006

Recommended for Publication by Ming Jiang

Based on an independent forward model in fluorescent tomography, a parallel reconstructed scheme for inhomogeneous mediums with unknown absorption property is proposed in this paper. The method considers the two diffusion equations as separately describing the propagation of excited light in tissues with and without fluorescent probes inside. Then the concentration of fluorophores is obtained directly through the difference between two estimations of absorption coefficient which can be parallel inversed. In this way, the multiparameter estimation problem in fluorescent tomography is transformed into two independent single-coefficient determined schemes of diffusion optical tomography (DOT). Any algorithms proved to be efficient and effective in DOT can be directly applied here. In this study the absorption property is estimated from the independent diffusion equations by a gradient-based optimization method with finite element method (FEM) solving the forward model. Simulation results of three representative occasions show that the reconstructed method can well estimate fluorescent property and tissue absorption distribution.

Copyright © 2006 Xiaolei Song et al. This is an open access article distributed under the Creative Commons Attribution License, which permits unrestricted use, distribution, and reproduction in any medium, provided the original work is properly cited.

### 1. INTRODUCTION

The mighty advance in biocompatible, specific fluorescent probes, combining with progress in photonic technology and methodology of modeling photon propagation, greatly promoted the development of fluorescence tomography in recent years. By transporting the ways gene expression and protein function visualized in vivo, fluorescence tomography becomes a promising means for molecularly based noninvasive imaging of biological tissues [1, 2].

Always fluorescent beacons emitting in the near infrared (NIR) bandwidth are preferred, since in this spectral window hemoglobin and water absorb minimally so as to allow photons to penetrate for several centimeters in tissues. In continuous wave (CW) mode, the following coupled diffusion equations are always employed to describe the propagation of both excitation and fluorescent emission light in diffusive medium [3–5]:

$$\nabla D_x(\mathbf{r})\nabla\Phi_x(\mathbf{r}) - [\mu_{a_x}(\mathbf{r}) + \mu_{a_f}(\mathbf{r})]\Phi_x(\mathbf{r}) = -\Theta_s\delta(\mathbf{r} - \mathbf{r}_{s_k}), \tag{1a}$$

$$\nabla D_m(\mathbf{r})\nabla\Phi_m(\mathbf{r}) - \mu_{a_m}(\mathbf{r})\Phi_m(\mathbf{r}) = -\Phi_x(\mathbf{r})\eta\mu_{a_f}(\mathbf{r}), \tag{1b}$$

where $\Phi_{x,m}$ is the photon density for excitation (subscript $x$) or fluorescent light (subscript $m$), $D_{x,m}(\mathbf{r})$ is the diffusion coefficient, and $\mu_{a_{x,m}}(\mathbf{r})$ is the absorption coefficient of the tissue. The absorption of excite light due to fluorophores is described as $\mu_{a_f}(\mathbf{r})$ and the fluorescent yield $\eta\mu_{af}(\mathbf{r})$ is the required fluorescence parameter. Based on this model, several simulations with 3-dimensional and 2-dimensional geometries have been done using inversion approaches such as Born approximation employing Green's function [6, 7], Newton's or Newton-type optimization methods [3, 4] and disturbed method [8]. However, these studies only deal with reconstruction of fluorescence properties in homogeneous mediums or under the assumption of known background optical properties. For inhomogeneous mediums with unknown absorption property as in many practical cases, only A. B. Milstein et al. reconstructed fluorescent yield and lifetime as well as optical properties in frequency mode using nonlinear Bayesian method [5]. However, in their work the fluorescent properties could only be estimated from (1b) after the optical coefficients are determined. As the two inversion parts must be executed serially, the total reconstruction process is very time consuming.

A recently developed model makes the two diffusion equations independent and speeds up the forward model execution [9]. Based on this model, we proposed a new parallel reconstructed scheme for inhomogeneous mediums with unknown absorption property. In this study the absorption property is separately inversed from the independent diffusion equations by a gradient-based optimization method with finite element method (FEM) solving the forward model. Simulation results of three different occasions show that the reconstructed method can well estimate fluorophore concentration and tissue absorption distribution.

### 2. METHODOLOGY

### 2.1. Independent forward model

It can be easily seen that the two equations in (1) are originally dependent. For example, in (1a) the fluorescent probe acts as an absorptive heterogeneity to excited light with absorption coefficient $\mu_{a_f}(\mathbf{r})$, which equals $\varepsilon_x c(\mathbf{r})$. Here $c(\mathbf{r})$ is the spatially varying fluorophore concentration and $\varepsilon_x$ is the molar extinction coefficient and is always assumed constant. In the right items of (1b) the strength of fluorescent source is influenced by the excited photon flux at that position, which mainly depends on the incident excited light and tissue's optical property at the excitation wavelength. The fluorophores absorb the excited light and then emit fluorescent light with the probability of $\eta$, the quantum yield, which depends on the type of fluorophore and the chemical environment [5].

In CW mode the diffusive coefficient is always considered spatially invariant and known in advance as $D_{x,m}$. For fluorescent beacons whose excited and emission light are both in the near infrared spectral window, the Stokes shift is relatively small and the optical properties of the two wavelengths are similar, such as in human breast tissue [10, 11]. Thus the optical coefficients for both wavelengths $\lambda_x$ and $\lambda_m$ can be considered equal, such as in [5, 6], that is, $D_x = D_m = D$ and $\mu_{a_x} = \mu_{a_m} = \mu_a$. Based on above assumptions, R. B. Schulz et al. [9] proposed an independent forward model. Firstly they rewrite (1) as

$$\nabla D \nabla \Phi_x(\mathbf{r}) - [\mu_a(\mathbf{r}) + \varepsilon_x c(\mathbf{r})]\Phi_x(\mathbf{r}) = -\Theta_s \delta(\mathbf{r} - \mathbf{r}_{s_k}), \tag{2a}$$

$$\nabla D \nabla \Phi_m(\mathbf{r}) - \mu_a(\mathbf{r})\Phi_m(\mathbf{r}) = -\Phi_x(\mathbf{r})\eta \cdot \varepsilon_x c(\mathbf{r}). \tag{2b}$$

Then (2a) is converted to the following form:

$$\varepsilon_x c(\mathbf{r})\Phi_x(\mathbf{r}) = \Theta_s \delta(\mathbf{r} - \mathbf{r}_{s_k}) - (-\nabla D \nabla + \mu_a(\mathbf{r}))\Phi_x(\mathbf{r}). \tag{3}$$

Substituting (3) in (2b) and dividing it by $\eta$ yield

$$(-\nabla D \nabla + \mu_a(\mathbf{r}) + \varepsilon_x c(\mathbf{r}))\Phi_x(\mathbf{r}) = \Theta_s \delta(\mathbf{r} - \mathbf{r}_{s_k}), \tag{4a}$$

$$(-\nabla D \nabla + \mu_a(\mathbf{r}))\left(\frac{1}{\eta}\Phi_m(\mathbf{r}) + \Phi_x(\mathbf{r})\right) = \Theta_s \delta(\mathbf{r} - \mathbf{r}_{s_k}). \tag{4b}$$

With respect to $\Phi_t = (1/\eta)\Phi_m + \Phi_x$, (4a) and (4b) are independent and can be solved simultaneously. A parallel FEM system was proposed in [9] which cuts down by one half the time of original forward problem solving.

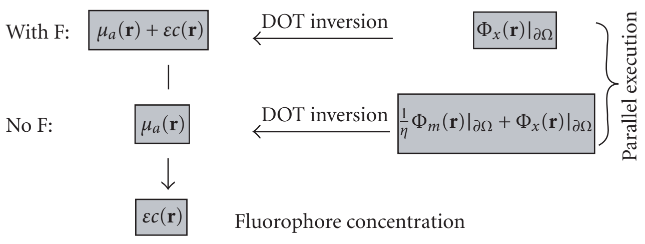

Figure 1: The process of parallel inversion scheme. The two "*F*" at the left are short for "fluorophore." The reconstruction of fluorescence tomography in heterogeneous medium is converted to two parallel absorption coefficient inversed problems in DOT.

### 2.2. Parallel inversion scheme

It can be easily found that the independent equations (4a) and (4b) have totally the same form as the single diffusion equation in diffuse optical tomography (DOT). The source items on the right side of equation are both the same $\delta$ functions and all the unknown properties are moved into the coefficient positions of the partial differential equations. Thus, for each equation, the estimation of unknown properties becomes a nonlinear reconstruction problem right as in DOT.

Equation (4) also has its physical significance, although educed through pure mathematic transform. Firstly (4a) describes the propagation of excitation light with the absorption of both tissue itself and the inside fluorophores. Then let us focus on (4b). The quantum yield, $\eta$, is always defined as a ratio between emitting fluorescence photon numbers and the number of excitation photons absorbed by fluorophore. Therefore, the item $(1/\eta)\Phi_m(r)$ can be treated as compensating the excitation photon density absorbed by fluorescent beacons and $\Phi_t$ shows the total excitation photon flux together with the fluorophore absorbed part. Thus, (4b) describes the transportation of excitation light in tissue with assumed no fluorochrome inside, providing a referenced absorption situation of only the tissue originating absorption $\mu_a(\mathbf{r})$ without fluorescent beacon administration.

Based on above analysis, (4a) and (4b) provide two different absorption situations of excitation wavelength with contrast caused by fluorescent beacon's absorption $\varepsilon_x c(\mathbf{r})$. Knowing that (4a) and (4b) have entirely the same form as absorption parameter inversed problems in DOT, we can parallel reconstruct $\mu_a(\mathbf{r}) + \varepsilon c(\mathbf{r})$ and $\mu_a(\mathbf{r})$ from the boundary measurement of excited light and fluorescent light according to the two independent diffusion equations. Then their contrast caused by fluorophore $\varepsilon_x c(\mathbf{r})$, which is proportional to the concentration of fluorescent probes, can be directly obtained by the difference of two absorption images. Figure 1 shows the process of the parallel reconstruction scheme.

So the parallel scheme converts the reconstruction of fluorescent property in heterogeneous medium to two independent absorption coefficient inversion problems in DOT. Both the unknown tissue absorption property and fluorophore concentration are determined. In this way, the multiparameter estimation problem in FODT is simplified to two single coefficient determined schemes of DOT. Any algorithms

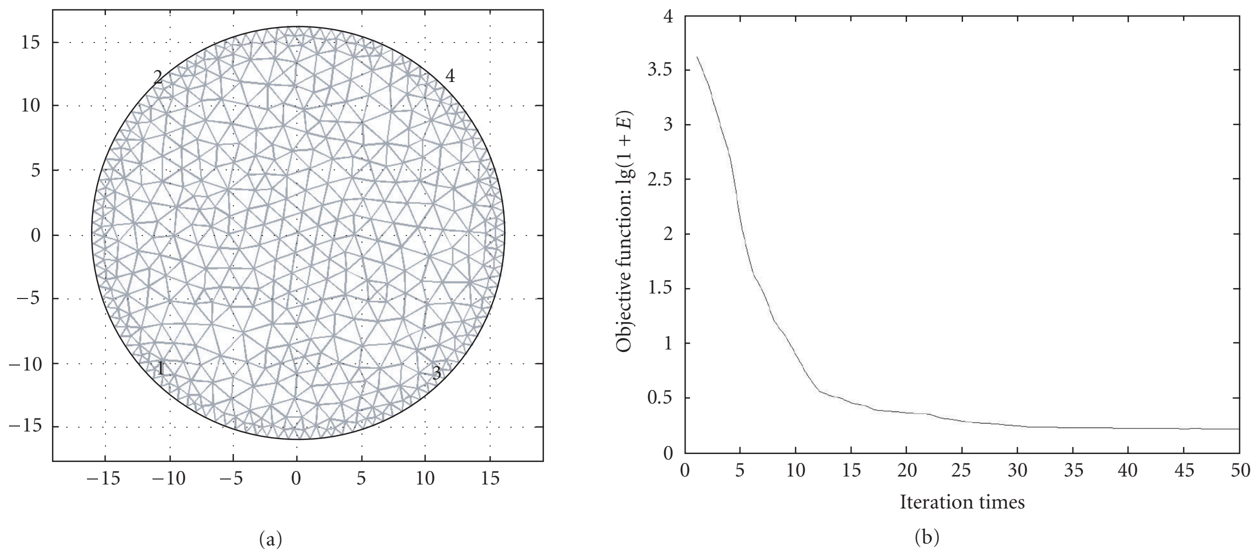

FIGURE 2: (a) shows the finite-element mesh used in the simulation. In (b), $X$ axis is the CG iteration number, $Y$ axis is the value of $\lg(1+E)$, representing the logarithm of objective function $E$.

proved to be efficient and effective in DOT can be directly applied here. Additionally, the total reconstructed time is the same as single DOT problem for which the two absorption property inversions can be performed at the same time.

### 2.3. Gradient-based optimization inversed method

To demonstrate the parallel reconstruction strategy, in this work a gradient-based optimization inversion method is used for the absorption coefficient inversion with FEM solving the forward model [12, 13]. Considering an experimental setting that includes $S$ point excitation light sources located at $\xi_j \in \partial\Omega$ $(j = 1,\dots,S)$, and $M_j$ measurement positions $\varsigma_{j,i} \in \partial\Omega$ $(i = 1,\dots,M_j)$ for each source $j$, the following objective function can be defined:

$$E = \frac{1}{2}\sum_{j=1}^{S}\sum_{i=1}^{M_j}\left((\Gamma_{j,i})_{\text{me}} - (\Gamma_{j,i})_c\right)^2, \quad (5)$$

where $\Gamma_{j,i}$ represents the photon intensity measured at position $\varsigma_{j,i}$ with the incident excitation source located at $\xi_j$. The subscript $c$ denotes the values calculated by the forward simulation and me represents the experimental (or in this case computer-simulated) values. By minimizing the objective function, we can handle an iteration computing and reconstruct the distribution of absorption coefficient.

To solve the optimization problem using a conjugate gradient (CG) method, the gradient of the objective function needs to be calculated as follows:

$$\nabla E = \sum_{j=1}^{S}\sum_{i=1}^{D_j}\left((\Gamma_{j,i})_{\text{me}} - (\Gamma_{j,i})_c\right)\cdot\left(-\frac{\partial(\Gamma_{j,i})_c}{\partial\mu_a}\right). \quad (6)$$

Therefore, the gradient vector $\vec{z}$ can be presented as

$$\vec{z} = \nabla E = -\mathbf{J}^T\mathbf{b}, \quad (7)$$

where $\mathbf{J}$ is an $M_{\text{TOT}}\times N_{\text{TOT}}$ Jacobian matrix, $M_{\text{TOT}} = \sum_{j=1}^{S} M_j$ is the total measurement number at the boundary, and $N_{\text{TOT}}$ is the number of the coefficients to be reconstructed. Here $\mathbf{b}$ is the residual error between the boundary measurements and computation values. Then $\mathbf{J}$ can be calculated by an adjoint source scheme based on the establishment of PMDF (photon measurement density function, as defined in [14]).

With the gradient calculated, the next step is to conduct one-dimensional search in order to find a best step length on this gradient direction. Then, we refresh the absorption coefficient and recalculate the gradient to form iteration computing until the error reaches the supposed value.

### 3. SIMULATION AND RESULTS

In this section, a group of simulation tests is done to demonstrate our parallel inversion scheme. For each reconstruction, three kinds of figures are produced, they are (1) absorption distribution due to both medium heterogeneity and extrinsic fluorescent probes, that is, $\mu_a(\mathbf{r}) + \varepsilon\mathbf{c}(\mathbf{r})$; (2) absorption coefficient distribution $\mu_a(\mathbf{r})$ of the heterogeneous medium without fluorophore; (3) $\varepsilon\mathbf{c}(\mathbf{r})$, which is just the minus result of the former two absorption coefficient distributions, representing the fluorochrome concentration as $\varepsilon$ is always assumed to be constant.

In our simulation, the imaging area is a circle of 32 mm diameter with the Robin boundary condition $\Phi(\xi) + 2D\cdot A\mathbf{n}\nabla\Phi(\xi) = 0$, $\xi \in \partial\Omega$, applied [15]. For both the excitation and emission wavelengths, the diffusion parameter of the medium is assumed constant and known as $D = 1/3[\mu_a+\mu_s']$,

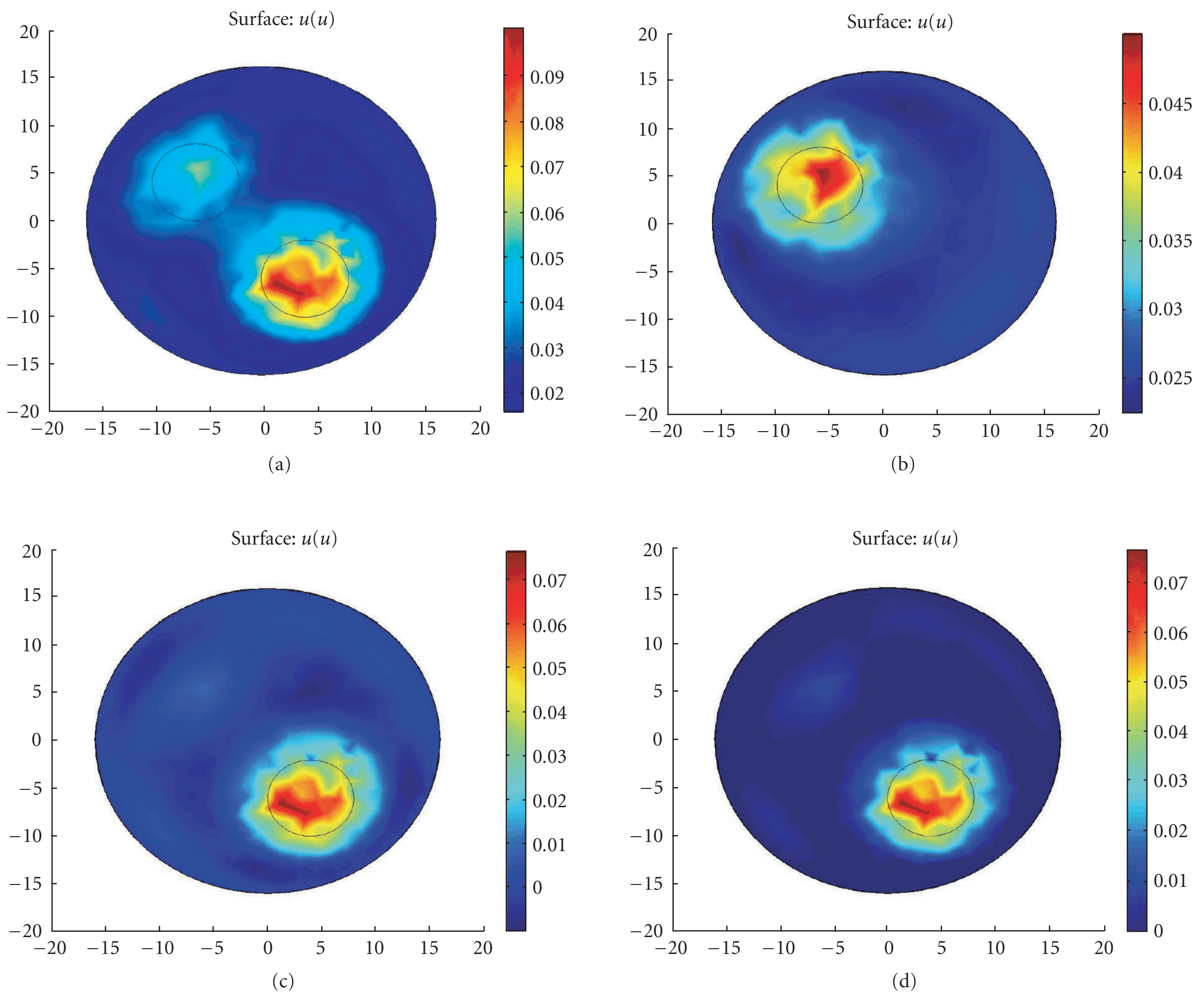

FIGURE 3: Images obtained in the case when FA and HA are apart from each other. (a) and (b) are, respectively, the independent reconstructed images of $\mu_a(\mathbf{r}) + \varepsilon\mathbf{c}(\mathbf{r})$ and $\mu_a(\mathbf{r})$. (a) minus (b) educed (c), describing the concentration of fluorochome. (d) constrains the negative values in (c) to zero. The black circles represent the original exact locations and shapes of HA and FA. The original $\mu_a(\mathbf{r})$ is 0.05 mm$^{-1}$ in the left 8 mm-diameter circle centred at $(-6, 4)$ and is 0.025 mm$^{-1}$ in the rest. $\varepsilon\mathbf{c}(\mathbf{r})$ is 0.075 mm$^{-1}$ in the right 8 mm-diameter circle centred at $(4, -6)$ and is zero in the rest.

with the reduced scattering coefficient $\mu_s' = 2.0\,\text{mm}^{-1}$ and the homogenous background absorption coefficient $\mu_a = 0.025\,\text{mm}^{-1}$. The 32 excited sources and 32 detectors are positioned alternately around the circle with equal intervals between each other. As the sources are collimated beams, they are represented by isotropic point sources located at one diffusion length of $1/\mu_s'$ below the border [15].

The forward model was solved by the FEMLAB2.1 (COMSOL Inc., Burlington, MA 01803, USA, a FEM solution in MATLAB (The Math Works, Inc.)), and all the codes are programmed in MATLAB6.0. The finite-element mesh for reconstruction consists of 544 nodes and 966 triangle elements (Figure 2(a)). The final estimated images reported in the paper are all of 25 CG iterations as the objective function descends very slowly when the iterative time is more than 25 (Figure 2(b)).

The simulated boundary measurement data $\Phi_x(\xi)$ and $\Phi_m(\xi)$ were generated separately from the forward model of (4a) and (4b) with exact distributions of $\mu_a(\mathbf{r})$ and $\varepsilon\mathbf{c}(\mathbf{r})$. According to different positions of the fluorochrome with respect to original absorption heterogeneity of tissue, we reconstructed the following three kinds of occasions in noise-free environment. (Here we abbreviate the original absorption heterogeneity as HA and absorption due to fluorescent probes as FA), that is, 1.HA and FA are apart from each other; 2.HA and FA intersect; 3.FA is within HA. We will present these results one by one.

Figures 3, 4, 5 separately show the reconstructed images produced by the parallel scheme, in which (a) and (b) are the reconstructed distributions of $\mu_a(\mathbf{r}) + \varepsilon\mathbf{c}(\mathbf{r})$ and $\mu_a(\mathbf{r})$, respectively. And (a) minus (b) results in (c), describing the concentration of fluorochome. Due to the differences in

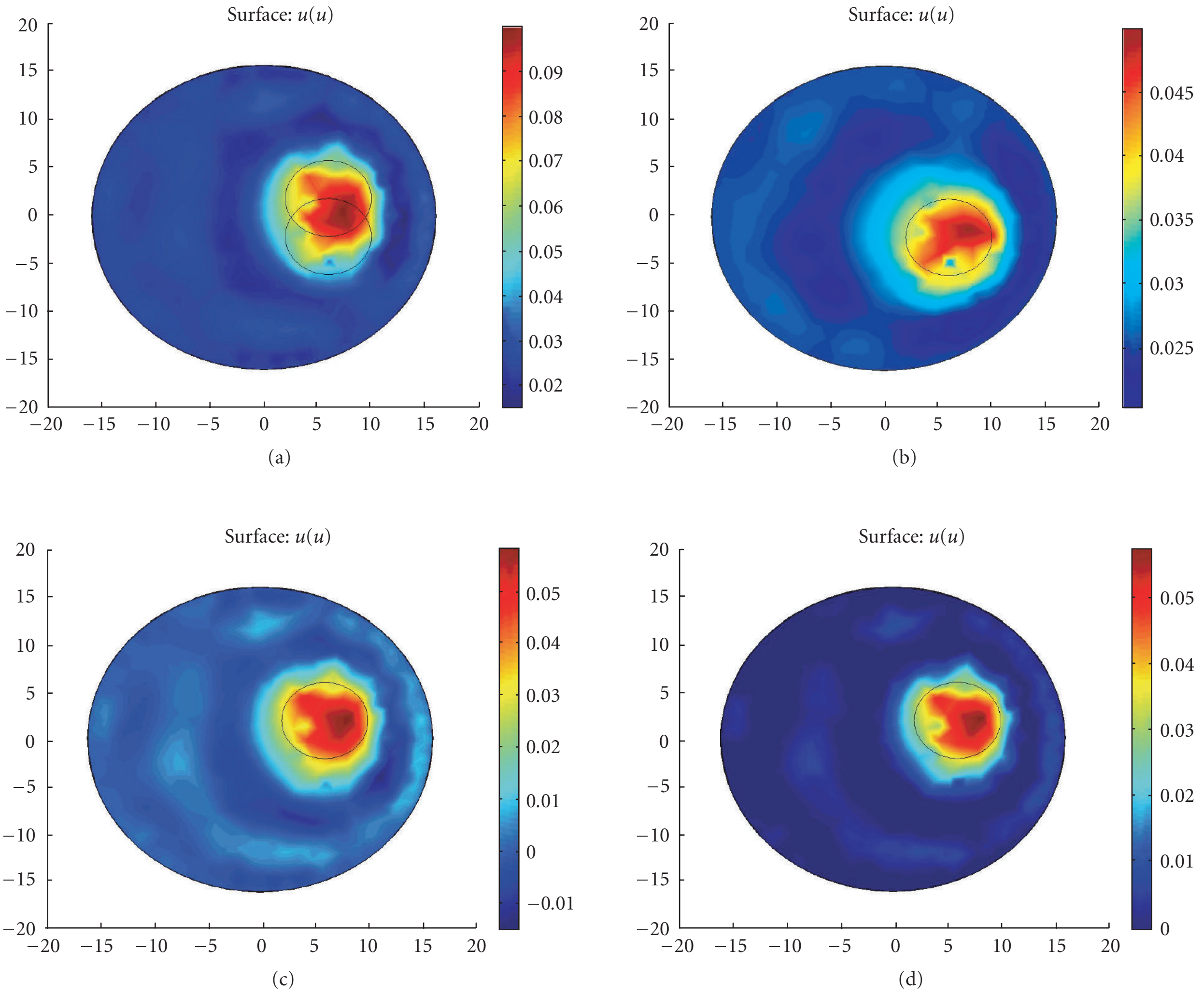

FIGURE 4: Images reconstructed when FA and HA intersect. (a) and (b) are, respectively, the independent reconstructed images of $\mu_a(\mathbf{r})+\varepsilon\mathbf{c}(\mathbf{r})$ and $\mu_a(\mathbf{r})$. (a) minus (b) educed (c), describing the concentration of fluorochome. (d) constrained the negative values in (c) to zero. The black circles represent the original exact locations and shapes of HA and FA. The original $\mu_a(\mathbf{r})$ is 0.05 $mm^{-1}$ in the lower 8 mm-diameter circle centred at (6, −2) and is 0.025 $mm^{-1}$ in the rest. $\varepsilon\mathbf{c}(\mathbf{r})$ is 0.05 $mm^{-1}$ in the upper 8 mm-diameter circle centred at (6,2) and is zero in the rest. Thus, the total absorption in the overlapped part is 0.10 $mm^{-1}$ in (a).

reconstructed qualities of the two dependent inverse problems, the minus result (c) will have values less than zero. However it is known that the concentration should not be negative in practice. So that these negative values are constrained to zero in our simulation. The modified results with nonnegative restriction in (d) show less background noise and better reconstructed qualities compared with the unconstrained ones of (c) in each figure.

### 4. DISCUSSION AND CONCLUSIONS

In last section, through the parallel inversion scheme, we reconstructed the three different relative distributions of tissue absorption heterogeneity and fluorophore in 2-dimensional geometry, combined with a nonnegative restriction. Distributions of fluorophore concentration as well as the absorption properties of tissue is obtained, by treating fluorescent tomography in inhomogeneous tissues as two independent nonlinear estimations in DOT. Although reconstruction of fluorophore concentration is a linear problem when the optical properties are known in advance, the parallel method has its superiorities. Firstly the two nonlinear estimations can be executed synchronously, so the total reconstruction time keeps the same as single absorption inversion in DOT. Then, any reconstruction algorithms which are already proved to be effective and efficient in DOT can be directly applied here. In this paper a basic CG method is employed for the reconstruction of absorption coefficient with noise-free data. However, for noisy data, the spatial resolution and the ability to solve deep located heterogeneities are very limited with only the

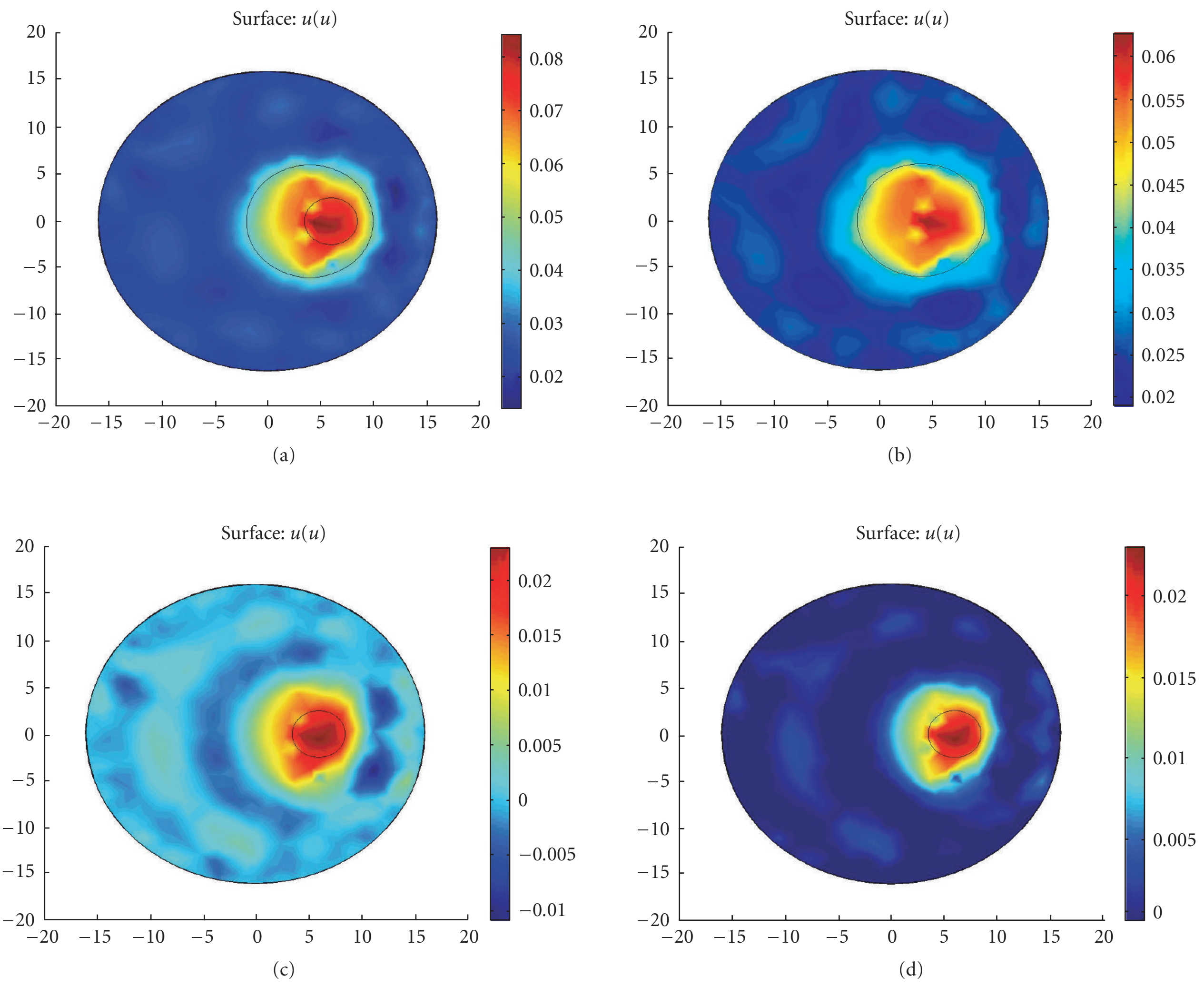

FIGURE 5: Images inversed when FA and HA intersect. (a) and (b) are, respectively, the independent reconstructed images of $\mu_a(\mathbf{r})+\varepsilon\mathbf{c}(\mathbf{r})$ and $\mu_a(\mathbf{r})$. (a) minus (b) educed (c), describing the concentration of fluorochome. (d) constrained the negative values in (c) to zero. The black circles represent the original exact locations and shapes of HA and FA. The original $\mu_a(\mathbf{r})$ is 0.05 $\text{mm}^{-1}$ in the bigger 12 mm-diameter circle centred at $(4, 0)$ and is 0.025 $\text{mm}^{-1}$ in the rest. $\varepsilon\mathbf{c}(\mathbf{r})$ is 0.05 $\text{mm}^{-1}$ in the inside 5 mm-diameter circle centred at $(6, 0)$ and is zero in the rest.

basic CG method [13]. Other strategies can be added to improve the reconstructed image quality such as the spatial located weighted method [13] and adaptive finite element technology [16]. Additionally, it does not require measurements of excitation light before fluorochrome administration for estimating the absorption property of tissue, as $\Phi_t$ in (4b) could provide an absolute photon-field measurement.

In the independent model and the parellel reconstruciton method, a constant quantum yield is a basic assumption of quantitative fluorescence imaging as in [5, 17]. In fact, it might be questionable in biological environments. However, newly developed emitters like quantum dots which are embedded in an isolator are independent of the environment [18]. Therefore, it offers a practical and relative simple method to determine fluorescent concentration in heterogeneous medium and tissues, as $\Phi_t$ can be easily combined from boundary measurements $\Phi_x$, $\Phi_m$ using the optics filter.

### ACKNOWLEDGMENTS

This work is supported in part by the National Natural Science Foundation of China, the Tsinghua-Yue-Yuen Medical Science Foundation, the National Basic Research Program of China, and the Special Research Fund for the Doctoral Program of Higher Education of China.

### REFERENCES

[1] V. Ntziachristos, J. Ripoll, L. V. Wang, and R. Weissleder, "Looking and listening to light: the evolution of whole-body photonic imaging," *Nature Biotechnology*, vol. 23, no. 3, pp. 313–320, 2005.

[2] V. Ntziachristos, C. Bremer, and R. Weissleder, "Fluorescence imaging with near-infrared light: new technological advances that enable in vivo molecular imaging," *European Radiology*, vol. 13, no. 1, pp. 195–208, 2003.

[3] H. Jiang, "Frequency-domain fluorescent diffusion tomography: a finite-element-based algorithm and simulations," *Applied Optics*, vol. 37, no. 22, pp. 5337–5343, 1998.

[4] D. Y. Paithankar, A. U. Chen, B. W. Pogue, M. S. Patterson, and E. M. Sevick-Muraca, "Imaging of fluorescent yield and lifetime from multiply scattered light reemitted from random media," *Applied Optics*, vol. 36, no. 10, pp. 2260–2272, 1997.

[5] A. B. Milstein, S. Oh, K. J. Webb, et al., "Fluorescence optical diffusion tomography," *Applied Optics*, vol. 42, no. 16, pp. 3081–3094, 2003.

[6] M. A. O'Leary, D. A. Boas, X. D. Li, B. Chance, and A. G. Yodh, "Fluorescence lifetime imaging in turbid media," *Optics Letters*, vol. 21, no. 2, pp. 158–160, 1996.

[7] V. Ntziachristos and R. Weissleder, "Experimental three-dimensional fluorescence reconstruction of diffuse media by use of a normalized Born approximation," *Optics Letters*, vol. 26, no. 12, pp. 893–895, 2001.

[8] E. L. Hull, M. G. Nichols, and T. H. Foster, "Localization of luminescent inhomogeneities in turbid media with spatially resolved measurements of cw diffuse luminescence emittance," *Applied Optics*, vol. 37, no. 13, pp. 2755–2765, 1998.

[9] R. B. Schulz, J. Peter, W. Semmler, and W. Bangerth, "Independent modeling of fluorescence excitation and emission with the finite element method," in *Proceedings of OSA Biomedical Topical Meetings*, Miami, Fla, USA, April 2004.

[10] M. J. Eppstein, F. Fedele, J. Laible, C. Zhang, A. Godavarty, and E. M. Sevick-Muraca, "A comparison of exact and approximate adjoint sensitivities in fluorescence tomography," *IEEE Transactions on Medical Imaging*, vol. 22, no. 10, pp. 1215–1223, 2003.

[11] B. J. Tromberg, O. Coquoz, J. B. Fishkin, et al., "Non-invasive measurements of breast tissue optical properties using frequency-domain photon migration," *Philosophical Transactions of the Royal Society of London Series B Biological Sciences*, vol. 352, no. 1354, pp. 661–668, 1997.

[12] S. R. Arridge and M. Schweiger, "A gradient-based optimisation scheme for optical tomography," *Optics Express*, vol. 2, no. 6, pp. 213–226, 1998.

[13] J. Zhou, J. Bai, and P. He, "Spatial location weighted optimization scheme for DC optical tomography," *Optics Express*, vol. 11, no. 2, pp. 141–150, 2003.

[14] S. R. Arridge and M. Schweiger, "Photon-measurement density functions. Part 2: finite-element-method calculations," *Applied Optics*, vol. 34, no. 34, pp. 8026–8037, 1995.

[15] M. Schweiger, S. R. Arridge, M. Hiraoka, and D. T. Delpy, "The finite element method for the propagation of light in scattering media: boundary and source conditions," *Medical Physics*, vol. 22, no. 11, pp. 1779–1792, 1995.

[16] A. Joshi, W. Bangerth, and E. M. Sevick-Muraca, "Adaptive finite element based tomography for fluorescence optical imaging in tissue," *Optics Express*, vol. 12, no. 22, pp. 5402–5417, 2004.

[17] E. E. Graves, J. Ripoll, R. Weissleder, and V. Ntziachristos, "A submillimeter resolution fluorescence molecular imaging system for small animal imaging," *Medical Physics*, vol. 30, no. 5, pp. 901–911, 2003.

[18] J. K. Jaiswal, H. Mattoussi, J. M. Mauro, and S. M. Simon, "Long-term multiple color imaging of live cells using quantum dot bioconjugates," *Nature Biotechnology*, vol. 21, no. 1, pp. 47–51, 2003.

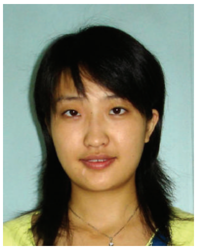

**Xiaolei Song** was born in 1982 in Mizhi, Shaanxi, China. She earned the B.S. degree from the Department of Biomedical Engineering in Xi'an Jiaotong University, Xi'an, Shaanxi, China in 2003. Then she was accepted as a Ph.D candidate by Department of Biomedical Engineering, Tsinghua University, Beijing, China. Now she is in the fourth year of her Ph.D studies. Her research work is mainly focused on Fluorescence Molecular Tomography and Diffusion Optical Tomography, especially the reconstruction.

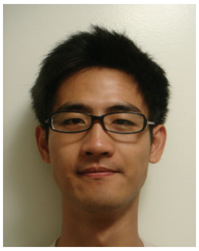

**Ji Yi** was born in 1982 in Anxi, Quanzhou, Fujian, China. He earned the B.S. in the Department of Biomedical Engineering, Tsinghua University. Now he is a Ph.D candidate in Northwest University, Chicago, USA.

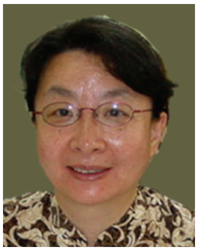

**Jing Bai** obtained the M.S. and Ph.D. degrees from Drexel University, Philadelphia, in 1983 and 1985. From 1985 to 1987 she was a Research Associate and Assistant Professor with the Biomedical Engineering and Science Institute of Drexel University. In 1988, 1991, and 2000 she become an Associate Professor, Professor, and Cheung Kong Chair Professor at Electrical Engineering Department of Tsinghua University, Beijing, China. Her research activities have included mathematical modeling and simulation of cardiovascular system, optimization of cardiac assist devices, medical ultrasound, telemedicine, home health care network and home monitoring devices, and Infrared imaging. She has published four books and over 100 journal papers. She is a Fellow of IEEE. From 1997, she becomes associate editor for IEEE Trans on Information Technology in Biomedicine.

